# Mass spectrometric detection of iron nitrosyls, sulfide oxidation and mycothiolation during nitrosylation of the NO sensor [4Fe–4S] NsrR†
†Electronic supplementary information (ESI) available. See DOI: 10.1039/c8cc01339j


**DOI:** 10.1039/c8cc01339j

**Published:** 2018-05-23

**Authors:** Jason C. Crack, Chris J. Hamilton, Nick E. Le Brun

**Affiliations:** a Centre for Molecular and Structural Biochemistry , School of Chemistry , University of East Anglia , Norwich Research Park , Norwich , NR4 7TJ , UK . Email: n.le-brun@uea.ac.uk; b School of Pharmacy , University of East Anglia , Norwich Research Park , Norwich , NR4 7TJ , UK

## Abstract

The bacterial nitric oxide (NO)-sensing transcriptional regulator NsrR binds a [4Fe–4S] cluster that enables DNA-binding and thus repression of the cell's NO stress response. Upon exposure to NO, the cluster undergoes a complex nitrosylation reaction resulting in a mixture of iron-nitrosyl species, which spectroscopic studies have indicated are similar to well characterized low molecular weight dinitrosyl iron complex (DNIC), Roussin's Red Ester (RRE) and Roussin's Black Salt (RBS). Here we report mass spectrometric studies that enable the unambiguous identification of NsrR-bound RRE-type species, including a persulfide bound form that results from the oxidation of cluster sulfide. In the presence of the low molecular weight thiols glutathione and mycothiol, glutathionylated and mycothiolated forms of NsrR were readily formed.

## Abstract 2

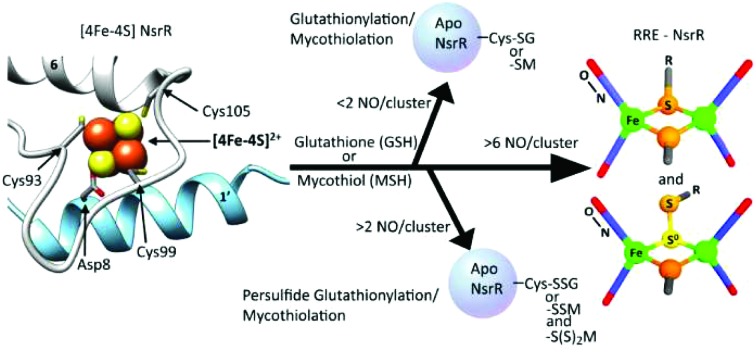
Identification of RRE-type iron-nitrosyl species formed upon nitrosylation of [4Fe–4S] NsrR.

## 

Proteins that contain iron–sulfur (Fe–S) clusters play key roles in an array of biochemical processes from respiration and photosynthesis to DNA replication. The clusters are generally fragile and are particularly reactive towards nitric oxide (NO),^1^ which is generated in macrophages during the initial immune response against invasion by microbes.^2^ Therefore, it is likely that Fe–S proteins are primary targets of NO at high (cytotoxic) concentrations, and perhaps also at lower (signalling) concentrations. Indeed, detection of paramagnetic dinitrosyl iron complexes (DNICs; [Fe(i)(NO)_2_(SR)_2_]^–^ in which RS^–^ are protein cysteinates) by EPR^3^–^6^ gave the first evidence of the *in vivo* reactivity of NO, and at least some of these species contained iron that was originally part of an Fe–S cluster.

Several bacterial NO sensor proteins, including NsrR and WhiB-like (Wbl) family proteins, contain an Fe–S cluster.^1^,^7^,^8^ NsrR, a [4Fe–4S] cluster-containing member of the Rrf2 family of transcriptional regulators, functions as part of the cell's response to nitrosative stress.^9^–^12^ Recently, the structure of the [4Fe–4S] cluster bound form of NsrR was solved, the first for any Fe–S cluster Rrf2 family member.^13^

The overall structure of NsrR (Fig. 1) is similar to that of apo-IscR^14^,^15^ and CymR,^16^ consisting of an elongated protein fold with a DNA-binding domain comprising four α-helices and two anti-parallel β-strands (α_1_, α_2_, α_3_, β_1_, β_2_, α_4_), which includes a winged helix-turn-helix motif. This is connected to a dimerization helix (α_6_, α_7_) *via* a loop containing three Cys residues that are conserved in IscR and other Fe–S cluster containing Rrf2 proteins. The cluster is coordinated by the three conserved Cys residues (Cys93, 99 and 105) and Asp8 from the other subunit of the NsrR dimer. The [4Fe–4S] NsrR structure, along with that of apo-NsrR, revealed the structural basis for cluster-dependent DNA binding.^13^ A network of H-bonds connects the Cys-containing cluster binding loop with the DNA recognition helix of the other subunit; loss of the cluster results in a less ordered cluster loop and partial loss of the H-bond network, resulting in a relative shift of the recognition helix.

**Fig. 1:**
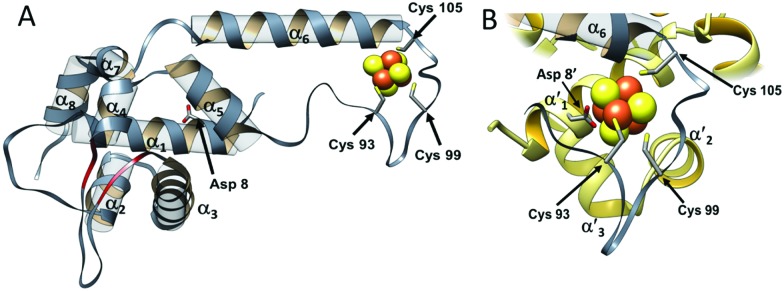
The structure of *S. coelicolor* [4Fe–4S] NsrR. (A) Ribbon depiction of one of the subunits of the NsrR dimer. α-Helices are labelled (α_1_–α_7_) and strands β_1_ and β_2_ are shown in red. Fe and S atoms are shown in brown and yellow, respectively. The locations of the Cys ligands (residues 93, 99, and 105) are shown. (B) The [4Fe–4S] cluster binding loop in more detail. The fourth ligand, Asp8, originates from the α′1 helix of the opposite monomer (PDB: ; 5N07).^13^

In pathogens, resistance to host-generated nitrosative stress is essential in order to establish infection.^17^ Recent advances in purifying and handling sufficiently high concentrations of often extremely sensitive Fe–S proteins have facilitated the application of a range of biophysical techniques to study their reactions with NO. In some cases, the kinetics and thermodynamics of cluster nitrosylation have been investigated; *S. coelicolor* WhiD and NsrR,^12^,^18^ and *Escherichia coli* FNR^19^ have been shown to react rapidly with NO in a complex multi-step processes involving up to ∼8 NO molecules per cluster. These and other studies have revealed that paramagnetic DNIC species are, in general, not the major products of Fe–S cluster nitrosylation; instead, EPR silent multinuclear iron species that appear to be similar to the well known inorganic complexes Roussin's Red Ester (RRE; [Fe_2_(NO)_4_(SR)_2_]) and Roussin's Black Salt (RBS; [Fe_4_(NO)_7_(S)_3_])^20^ have been shown to be major products.^18^,^19^,^21^–^23^ In particular, recent studies of NsrR and WhiD using the iron-specific vibrational technique nuclear resonance vibrational spectroscopy (NRVS) revealed that nitrosylation results in a mixture of iron nitrosyl products, with RRE-like and RBS-like species as principal products, along with minor amounts of DNIC species.^24^

To gain further insight into the reaction of NsrR with NO, liquid chromatography electrospray ionisation mass spectrometry (LC-ESI-MS) was used to analyse [4Fe–4S] NsrR before and after addition of NO. The deconvoluted mass spectrum of NsrR, Fig. 2A, was dominated by the apo-NsrR peak at 15 953 Da (theoretical mass of 15 954 Da, Table S1, ESI†), as expected because the cluster cannot survive the acidic conditions of LC-MS sample preparation. Minor peaks at +32, +56 and occasionally +112 Da were also observed, corresponding to a low amount of a persulfide adduct, and one and two irons, respectively, most likely resulting from the loss of the cluster during sample preparation. Exposure to an excess of NO (8 NO per [4Fe–4S] cluster) prior to analysis by LC-MS resulted in a 2 Da decrease in the mass of the apo-protein to 15 951 Da, suggesting that NO-mediated oxidation of apo-NsrR, resulting in a disulfide bond, occurred upon reaction with NO (Fig. S1, ESI†). MS/MS analysis of digested NsrR revealed that the disulfide bond forms between Cys93 and Cys99 (Fig. S2, ESI†).

**Fig. 2:**
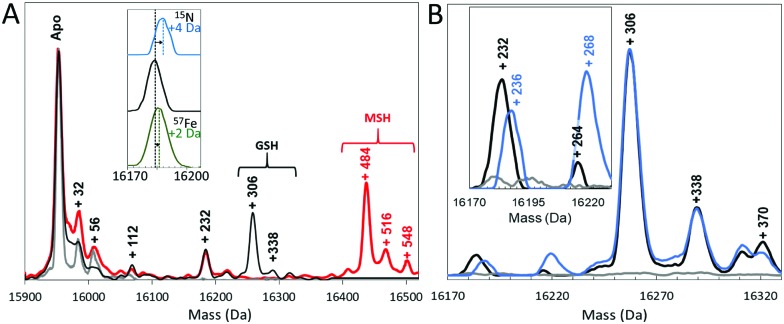
LC-MS of NsrR-bound iron-nitrosyls. (A) Deconvoluted LC-MS spectra of [4Fe–4S] NsrR before (grey line) and after exposure to nitric oxide in the presence of glutathione (GSH, black line) or mycothiol (MSH, red line). The major peak corresponds to apo-NsrR, with smaller peaks corresponding to adducts of S (+32 Da), Fe (+56 Da), GSH (+306 Da) and MSH (+484). Inset, isotopic labelling resulted in an isotope shift of the +232 Da peak ([Fe_2_(NO)_4_]) of +4 mass units in response to ^15^NO and +2 mass units in response to ^57^Fe. (B) NO induced LC-MS peaks (black line) corresponding to the addition of [Fe_2_(NO)_4_] (+232 Da), [Fe_2_(NO)_4_(S)] (+264 Da), GSH (+306 Da), and glutathione persulfide adducts (+338 and +370 Da). NO induced peaks obtained with ^15^NO (blue line) are in some cases shifted, revealing NO containing species. Inset, the lower mass region is shown in more detail.

In addition to this apo-protein mass shift, a number of additional peaks were observed at masses +232, +306, and +338 Da (relative to reduced apo-NsrR) upon reaction with NO, see Fig. 2A. Further low abundance peaks were also observed at +264 and +370 Da (Fig. 2B). The peak at +232 Da was consistent with the presence of an NsrR-bound RRE-type ([Fe_2_(NO)_4_]) species (Fig. 3A), while that at +306 Da corresponded to glutathionylated NsrR (NsrR covalently attached to a single glutathione, which was present in the protein sample buffer). Peaks at +338 and 370 Da could arise from glutathionylated NsrR with one and two additional sulfurs, respectively. The inclusion of glutathione was previously found to stabilize the solution against precipitation that occurred in the absence of a low molecular weight thiol at ratios of [NO] : [FeS] ≥ 2.^12^

**Fig. 3:**
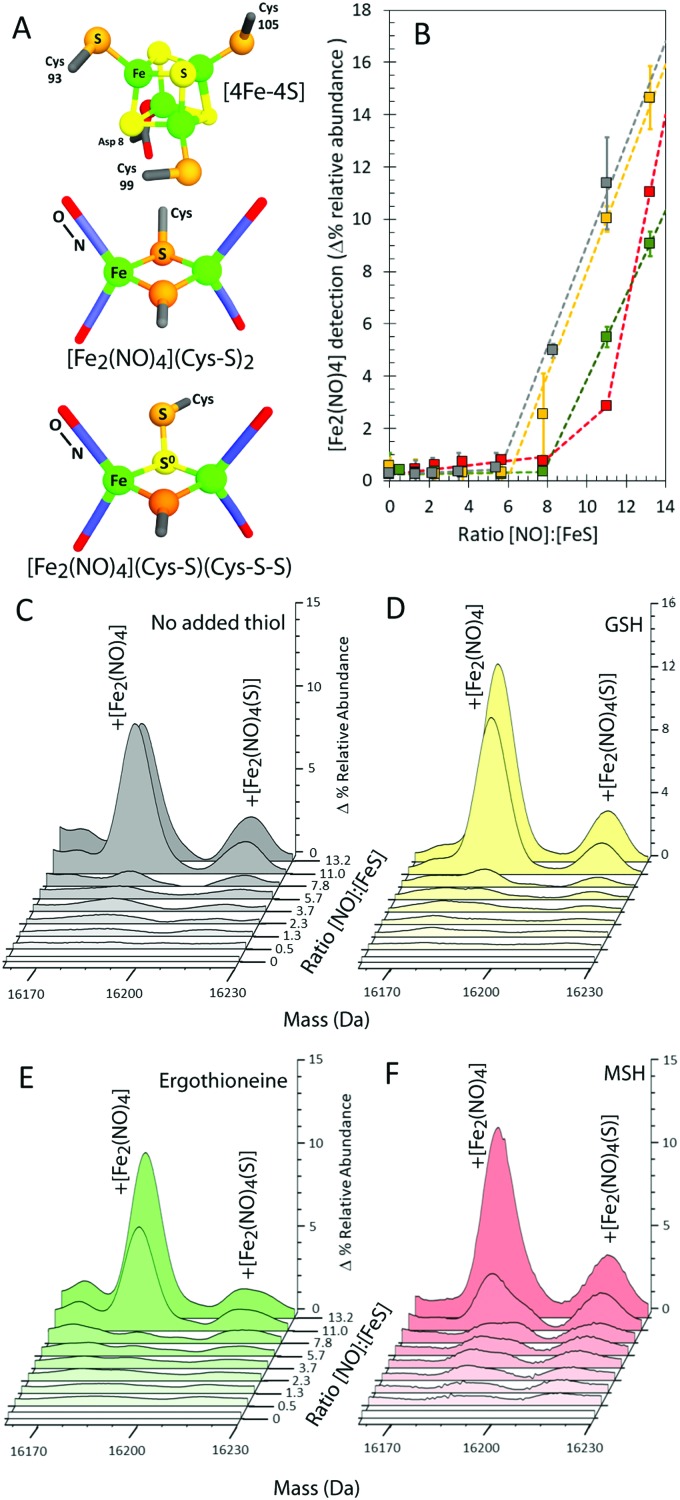
Formation of NsrR-bound RRE species. (A) Structures of the NsrR [4Fe–4S] cluster and proposed RRE and persulfide RRE species, where Cys is derived from the protein. (B) Formation of [Fe_2_(NO)_4_] adduct in the absence (grey circles) or presence of GSH (yellow squares), MSH (red squares) or ergothioneine (green squares). (C–F) LC-MS spectra of [4Fe–4S] NsrR following additions of NO in (C) the absence and presence of (D) glutathione (GSH), (E) ergothioneine, and (F) mycothiol (MSH). Samples were maintained under anaerobic conditions prior to LC-MS analysis. The data correspond to multiple repeats (*n* ≥ 2); error bars represent SEM.

To confirm these assignments and to help with those for other peaks, the reaction was repeated with ^57^Fe-substituted [4Fe–4S] NsrR and, separately, with ^15^NO. For the ^57^Fe NsrR experiment, the peak at +232 Da shifted to +234 Da, consistent with the two additional mass units associated with two ^57^Fe ions, see Fig. 2A. For ^15^NO, the peak at +232 Da shifted to +236 Da, consistent with the four additional mass units associated with four ^15^NO molecules (Fig. 2A and B). The peak at +264 Da was significantly increased in intensity and shifted to +268 Da in the ^15^NO spectrum, consistent with an RRE with one additional sulfur, *i.e.* a persulfide coordinated RRE, [[Fe_2_(NO)_4_](S)] (Fig. 3A). The peaks at +306, +338 and +370 Da were unaffected in the ^15^NO experiment (Fig. 2B), consistent with the absence of NO in glutathionylated NsrR containing zero, one and two additional sulfurs (*i.e.* NsrR-SG, NsrR-S-SG and NsrR-SS-SG), respectively.

To further investigate the NO-mediated oxidation of NsrR and the formation of RRE and glutathionylated species, similar experiments were performed with increasing levels of NO, both in the absence and presence of GSH. In both cases, the mass of the apo-NsrR peak varied systematically with increasing NO, decreasing by 2 Da in the absence of GSH and by ∼1 Da in the presence, demonstrating that GSH protects NsrR from oxidation (Fig. S1, ESI†). As above, peaks due to RRE iron-nitrosyl adducts of NsrR were observed to form, but these remained at very low abundance until the ratio NO : [4Fe–4S] was >6 in the presence and absence of GSH (Fig. 3B–D). This indicated that RRE species are products of cluster nitrosylation in a non-concerted reaction.^12^

In the presence of GSH, glutathionylated NsrR was formed initially linearly with NO, suggesting that the formation of the mixed disulfide results from NO-dependent oxidation (Fig. 4A and C). The glutathionylated products with one or two additional sulfurs formed later in the titration, appearing only after the addition of ∼2 NO per cluster (Fig. 4A and D). The source of these additional sulfurs must be the bridging sulfides from the Fe–S cluster and therefore the data indicate that sulfide is not released from the cluster and oxidized until >2 NO per cluster are added. This is consistent with recent DNA-binding experiments showing that binding of [4Fe–4S] NsrR to the *hmpA2* promoter (one of the three genes regulated by NsrR in *S. coelicolor*^9^) was abolished above a NO : [4Fe–4S] ratio of 2 : 1.^12^

**Fig. 4:**
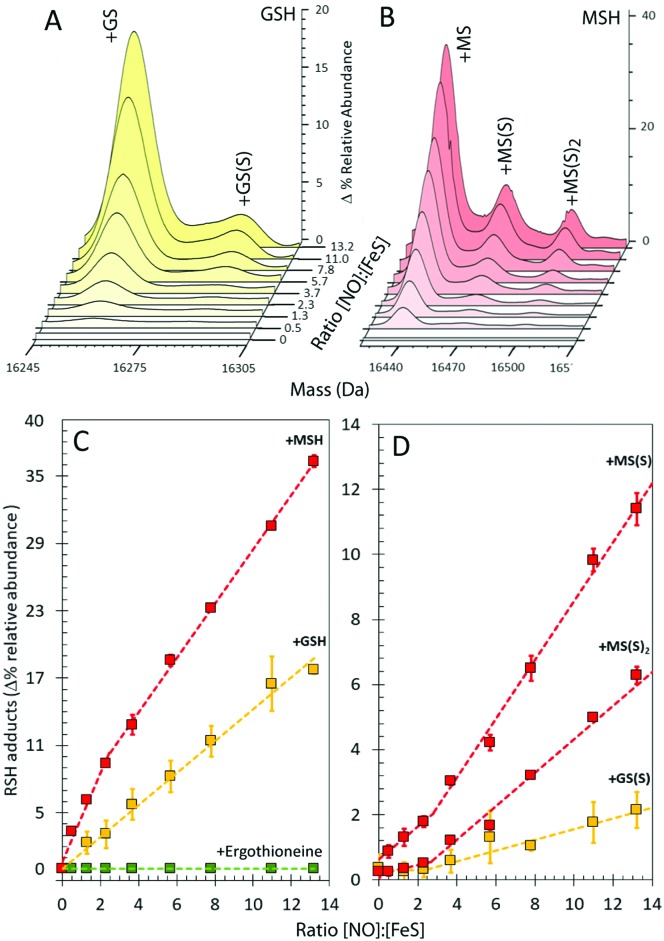
Formation of GSH and MSH adducts of NsrR. (A) LC-MS spectra of [4Fe–4S] NsrR following additions of NO in (A) glutathione (GSH) and (B) mycothiol (MSH). (C and D) NO dependent formation of thiol adducts of apo-NsrR (as indicated) (C) and persulfide thiol adducts (D). Samples/data analyses as in Fig. 3.

The cytoplasm of *S. coelicolor* does not contain GSH; instead, the low molecular weight thiols ergothioneine and mycothiol (MSH) are found, and so more physiologically relevant experiments were performed with ergothioneine and MSH.^25^,^26^ Like GSH, these protected NsrR from NO-mediated disulfide bond formation (Fig. S1C, ESI†). RRE iron-nitrosyl adducts of NsrR were observed, Fig. 3B, E and F, but these remained at very low abundance until the ratio NO : [4Fe–4S] was >8, indicating that ergothioneine/MSH inhibit the formation, or destabilize, NsrR-bound RREs. Mycothiolated NsrR was also observed, with the MSH adduct accumulating with increasing NO (Fig. 4B and C). The increase was more pronounced in the range 0–2 NO per cluster, compared to that above this ratio, and the extent of mycothiolation substantially greater than that observed for GSH. As for GSH, the formation of sulfur adducts of mycothiolated NsrR increased significantly above a ratio of 2 NO per cluster (Fig. 4D), again consistent with release/oxidation of cluster sulfide above this ratio. No covalent adducts were detected with ergothioneine (Fig. 4C). MS/MS analysis of digested NsrR revealed that GSH adducts are formed at Cys93 and Cys99 (see Fig. S3 and S4, ESI†), consistent with the structure of apo-NsrR, which indicates that these residues (but not Cys105) are surface exposed (Fig. S5, ESI†). Fragments containing Cys105 were not detected by MS/MS, so we cannot entirely rule out that GSH adducts are formed here also. Previous studies showed that nitrosylation of [4Fe–4S] NsrR in the presence of GSH resulted in complete loss of DNA binding,^12^ and so the function of thiol adducts is most likely the protection of Cys residues from NO-mediated oxidation of Cys93/Cys99 (Fig. S1 and S2, ESI†).

Recent studies of the reaction of NsrR with NO have provided clear evidence for the formation of RRE-like and RBS-like products, along with small amounts of DNIC species.^24^ That persulfide forms of these products might also be formed was postulated and DFT calculations supported this possibility. Along with the previous studies, the data presented here provide the first unequivocal proof that [Fe_2_(NO)_4_] and [[Fe_2_(NO)_4_](S)] adducts are formed following the NO reaction of NsrR. Persulfide adducts arise as a result of oxidation of cluster sulfide,^18^ and attachment of the resulting S^0^ species to Cys thiolate side chains, as observed following the O_2_ reaction of [4Fe–4S] FNR, the master anaerobic/aerobic metabolism regulator in *E. coli*.^27^,^28^ It is not clear why iron-nitrosyl species are able to remain bound to the protein under conditions of the LC-MS experiment, where non-covalent interactions are expected to be lost. Given that previous spectroscopic studies of these cluster nitrosylation reactions indicated that at least half the iron remained associated with the protein following gel filtration,^18^ a significant proportion of iron-nitrosyls must be lost under the conditions of the LC-MS experiment, as apo-protein is the most abundant species in the LC-MS spectra. Nevertheless, enough remains associated with the protein to permit detection and we suggest that this is a result of the relative chemical robustness of RRE compared to Fe–S clusters. RBS-type species, which were recently shown to be another major product of cluster nitrosylation in these proteins, apparently do not survive the conditions of the LC-MS experiment, possibly because any sulfide associated with such species would not survive the acidic conditions of sample preparation.^24^

We note that iron-nitrosyl species were previously observed by LC-MS attached to *Clostridium botulinum* HiPIP following reaction of the [4Fe–4S] cluster with NO.^29^ In that study, a peak at +232 Da was interpreted as arising from the association of two DNIC species. This was in part based on the observation of the typical DNIC *S* = ½ signal in the EPR spectrum. However, quantification of the EPR signal revealed sub-stoichiometric amounts of DNIC and so it is possible that both DNIC and RRE species were present. That a single DNIC adduct was not detected may suggest that the iron-nitrosyl detected was more likely to be the RRE species. Furthermore, in that study, additional peaks at +31–33 Da were also observed for apo-protein and iron-nitrosyl adducts, which were interpreted as representing nitrosated forms of the protein (predicted at +29–30 Da). Based on recent studies that have revealed the ease with which cluster sulfide is oxidized to form persulfide adducts^18^,^19^,^28^,^30^ it is perhaps more likely that these may represent persulfide (+32 Da) adducts. The iron-nitrosyl species observed here for NsrR are unlikely to be due to DNIC species because no single DNIC adduct was observed at +116 Da and it is known that DNIC species are only minor products of NsrR [4Fe–4S] cluster nitrosylation.^12^

In summary, here we have demonstrated the precise nature of the RRE-type species formed upon [4Fe–4S] cluster nitrosylation of NsrR as [Fe_2_(NO)_4_] and [[Fe_2_(NO)_4_](S)]. Reaction with NO also resulted in the formation of an oxidized (disulfide bonded) form of apo-NsrR, which was inhibited by the presence of low molecular weight thiols. The presence of GSH and MSH led to the formation of thiol adducts (representing glutathionylation and mycothiolation) at Cys93 and Cys99, while the presence of ergothioneine did not. Formation of thiol adducts most likely functions to protect Cys residues from NO-mediated oxidation that results in a disulfide bond between Cys93 and Cys99. Persulfide forms of GSH and MSH adducts, resulting from the release and oxidation of sulfide from the [4Fe–4S] cluster, were only observed to accumulate at significant levels at NO : [4Fe–4S] ratios >2, consistent with previous solution data showing the formation of a distinct intermediate at this ratio, and DNA-binding studies showing that NsrR dissociates from the *hmpA2* promoter at the same ratio.^12^ This indicates that once two NO molecules are bound to the [4Fe–4S] core, the cluster begins to disassemble, leading to release of sulfide that can participate in further reactions.

This work was supported by Biotechnology and Biological Sciences Research Council Grant BB/P006140/1 to NLB & JCC, by UEA through the purchase of the mass spectrometer used in this work, and by the FeSBioNet COST Action CA15133. We thank Dr Gerhard Saalbach, John Innes Centre (Proteomics Facility), for assistance in acquiring and analysing MS/MS data.

## Conflicts of interest

There are no conflicts to declare.

## Supplementary Material

Supplementary information

## References

[cit1] Crack J. C., Green J., Thomson A. J., Le Brun N. E. (2014). Acc. Chem. Res..

[cit2] Bruckdorfer R. (2005). Mol. Aspects Med..

[cit3] Vanin A. F., Kiladze S. V., Kubrina L. N. (1977). Biofizika.

[cit4] Drapier J. C. (1997). Methods.

[cit5] Vanin A. F. (2009). Nitric oxide.

[cit6] Costanzo S., Menage S., Purrello R., Bonomo R. P., Fontecave M. (2001). Inorg. Chim. Acta.

[cit7] Poole R. K. (2005). Biochem. Soc. Trans..

[cit8] Crack J. C., Green J., Thomson A. J., Le Brun N. E. (2012). Curr. Opin. Chem. Biol..

[cit9] Crack J. C., Munnoch J., Dodd E. L., Knowles F., Al Bassam M. M., Kamali S., Holland A. A., Cramer S. P., Hamilton C. J., Johnson M. K., Thomson A. J., Hutchings M. I., Le Brun N. E. (2015). J. Biol. Chem..

[cit10] Tucker N. P., Hicks M. G., Clarke T. A., Crack J. C., Chandra G., Le Brun N. E., Dixon R., Hutchings M. I. (2008). PLoS One.

[cit11] Yukl E. T., Elbaz M. A., Nakano M. M., Moenne-Loccoz P. (2008). Biochemistry.

[cit12] Crack J. C., Svistunenko D. A., Munnoch J., Thomson A. J., Hutchings M. I., Le Brun N. E. (2016). J. Biol. Chem..

[cit13] Volbeda A., Dodd E. L., Darnault C., Crack J. C., Renoux O., Hutchings M. I., Le Brun N. E., Fontecilla-Camps J. C. (2017). Nat. Commun..

[cit14] Rajagopalan S., Teter S. J., Zwart P. H., Brennan R. G., Phillips K. J., Kiley P. J. (2013). Nat. Struct. Mol. Biol..

[cit15] Santos J. A., Alonso-Garcia N., Macedo-Ribeiro S., Pereira P. J. (2014). Proc. Natl. Acad. Sci. U. S. A..

[cit16] Shepard W., Soutourina O., Courtois E., England P., Haouz A., Martin-Verstraete I. (2011). FEBS J..

[cit17] Tucker N. P., Le Brun N. E., Dixon R., Hutchings M. I. (2010). Trends Microbiol..

[cit18] Crack J. C., Smith L. J., Stapleton M. R., Peck J., Watmough N. J., Buttner M. J., Buxton R. S., Green J., Oganesyan V. S., Thomson A. J., Le Brun N. E. (2011). J. Am. Chem. Soc..

[cit19] Crack J. C., Stapleton M. R., Green J., Thomson A. J., Le Brun N. E. (2013). J. Biol. Chem..

[cit20] Butler A. R., Glidewell C., Li M. (1988). Adv. Inorg. Chem..

[cit21] Harrop T. C., Tonzetich Z. J., Reisner E., Lippard S. J. (2008). J. Am. Chem. Soc..

[cit22] Tinberg C. E., Tonzetich Z. J., Wang H., Do L. H., Yoda Y., Cramer S. P., Lippard S. J. (2010). J. Am. Chem. Soc..

[cit23] Tonzetich Z. J., Wang H., Mitra D., Tinberg C. E., Do L. H., Jenney, Jr. F. E., Adams M. W., Cramer S. P., Lippard S. J. (2010). J. Am. Chem. Soc..

[cit24] Serrano P. N., Wang H., Crack J. C., Prior C., Hutchings M. I., Thomson A. J., Kamali S., Yoda Y., Zhao J., Hu M. Y., Alp E. E., Oganesyan V. S., Le Brun N. E., Cramer S. P. (2016). Angew. Chem..

[cit25] Genghof D. S., Van Damme O. (1968). J. Bacteriol..

[cit26] Newton G. L., Buchmeier N., Fahey R. C. (2008). Microbiol. Mol. Biol. Rev..

[cit27] Zhang B., Crack J. C., Subramanian S., Green J., Thomson A. J., Le Brun N. E., Johnson M. K. (2012). Proc. Natl. Acad. Sci. U. S. A..

[cit28] Crack J. C., Thomson A. J., Le Brun N. E. (2017). Proc. Natl. Acad. Sci. U. S. A..

[cit29] Foster H. W., Cowan J. A. (1999). J. Am. Chem. Soc..

[cit30] Johnson K. A., Verhagen M. F. J. M., Brereton P. S., Adams M. W. W., Amster I. J. (2000). Anal. Chem..

