# Supplementary material for: Mass spectrometric detection of iron nitrosyls, sulfide oxidation and mycothiolation during nitrosylation of the NO sensor [4Fe–4S] NsrR
Source: Chem Commun (Camb). 2018 May 23;54(47):5992–5. doi: 10.1039/c8cc01339j (PMC5994877; doi:10.1039/c8cc01339j)
Supplement: Supplementary file 1 [file CC-054-C8CC01339J-s001.pdf]

Electronic Supplementary Material (ESI) for ChemComm.
This journal is © The Royal Society of Chemistry 2018

Electronic Supplementary Information

Mass spectrometric detection of iron nitrosyls, sulfide oxidation and mycothiolation during nitrosylation of the NO sensor [4Fe-4S] NsrR

Jason C. Crack, Chris J. Hamilton and Nick E. Le Brun

**Table of Contents**	**Page**
**Experimental section**	2
Table S1. Predicted and observed masses for different forms of NsrR.	4
Figure S1. NO dependent disulfide bond formation in apo-NsrR.	5
Figure S2. MS/MS analysis reveals the location of the disulfide bond of oxidised apo-NsrR.	6
Figure S3. MS/MS analysis of GSH adducts of apo-NsrR.	7
Figure S4. MS/MS analysis of a Cys99 GSH adduct of apo-NsrR.	8
Figure S5. Structure of apo-NsrR showing the positions of residues 93, 99 and 105.	9
**References**	10

Experimental Procedures

Protein purification. Non tagged NsrR was purified under anaerobic conditions as previously described.[1] Protein concentrations were determined by the methods of Smith (Pierce),[2] with bovine serum albumin as the standard. Cluster content was determined using an extinction coefficient at $\varepsilon_{406\ nm}$ of 13.30 (±0.19) mM^{-1} cm^{-1}.[1] ^{57}Fe substituted [4Fe-4S] NsrR was prepared as previously described,[3, 4]

Low molecular weight thiols. Mycothiol (1-O-[2[-[[(2R)--(acetylamino)-3mercapto-1-oxopropyl]amino]-2-deoxy-D-glucopyranoside]-D-*myo*-inositol, MSH) was prepared via mycothiol disulfide, as previously described,[5] reduced via immobilized TCEP gel (Pierce, ThermoFisher), and quantitated using a DTNB assay ($\varepsilon_{412\ nm}$ ≈14,150 M^{-1} cm^{-1} .[6] L-Ergothioneine and glutathione (GSH) were obtained from commercial sources (Enzo Life sciences, Sigma-Aldrich, respectively). MSH, GSH and ergothioneine solutions were prepared in assay buffer immediately before use.

Liquid chromatography mass spectroscopy of intact NsrR (LC-MS). A sample (~100 µM [4Fe-4S], 2 ml) of NsrR was titrated with varying aliquots of ProliNONOate (Cayman Chemicals, USA) in assay buffer (20 mM Bis-tris propane, 20 mM Tris, 20 mM MES, 100 mM NaCl, 5% (v/v) glycerol, pH 8.0) to achieve the desired [NO]:[[4Fe-4S] ratio. After each addition of ProliNONOate, the sample was incubated for 5 min prior to the removal of 50 µl aliquot, which was immediately diluted to ~10 µM with an aqueous mixture of 2% (v/v) acetonitrile, 0.1% (v/v) formic acid in an LC-MS vial. At the end of the titration, LC-MS samples were removed from the anaerobic cabinet and loaded onto a ProSwift RP-1S column (4.6 x 50 mm) (Thermo Scientific) using an Ultimate 3000 uHPLC system (Dionex, Leeds, UK). Bound proteins were eluted (0.2 ml/min) using a linear gradient (15 min) from 2% to 100% (v/v) acetonitrile, 0.1% (v/v) formic acid.

The eluent was continuously infused into a Bruker microQTOF-QIII mass spectrometer, running Hystar (Bruker Daltonics, Coventry, UK)), using positive mode electrospray ionisation (ESI). Compass Data Analysis 4.1, with Maximum Entropy v1.3 was used for processing of spectra under the LC peak. The mass spectrometer was calibrated with ESI-L tuning mix (Agilent Technologies, California, USA). Exact masses are reported from peak centroids representing the isotope average neutral mass. Predicted masses are given as the isotope average of the neutral protein or protein complex, in which cofactor-binding is expected to be charge compensated. Mass spectrometry data are presented in terms of relative abundance, with the most abundance peak assigned to 100% and all other peaks reported relative to it (% relative abundance). Where delta percentage relative abundance (Δ % relative abundance) is plotted, the starting [4Fe-4S] NsrR spectrum was substracted to remove the baseline in order to clearly show NO-dependent changes. Stock solutions of ProliNONOate were freshly prepared in 50 mM NaOH and quantitated by absorbance, $\varepsilon_{252\ nm}$= 8400 M^{-1} cm^{-1} before use. Where appropriate, the assay buffer also contained low molecular weight thiols, MSH (0.6 mM), ergothioneine (2.1 mM) and GSH (1.2 mM). For samples containing ^{15}N-nitric oxide, a 50 µl aliquot of [4Fe-4S] NsrR was placed in a 0.5 ml PCR tube and the head space flushed with 1 ml of ^{15}N-NO gas (Cambridge Isotope Laboratories Inc / CK Gas Products Ltd.) and the sample gently agitated at ambient temperature for 5 min. The head space of the sample was replaced a further two times.

Liquid chromatograph MS/MS of NsrR peptides. Digestion and MS/MS analysis of identical NsrR samples (nitrosylated at ~15 [NO]:[FeS]) was performed independently in one of two ways. In the first, aliquots of the protein samples (1 µg) were diluted into 30 µl of 25 mM sodium phosphate buffer (pH 7.2), 0.5% sodium deoxycholate (SDC). Sequencing grade trypsin or sequencing grade AspN proteases (Promega) were added and the samples incubated at 30 °C for 6 h. The digestions were stopped, and the SDC precipitated, by adding 1 volume of 1% (v/v) formic acid. Aliquots of the digested protein were used for LC-MS/MS analysis on an Orbitrap-Fusion™ mass spectrometer (Thermo Fisher, Hemel Hempstead, UK) equipped with an UltiMate™ 3000 RSLCnano System (Thermo Fisher) using a nanoEase M/Z column (HSS C18 T3, 100 Å, 1.8 µm; Waters, Wilmslow, UK). The samples were loaded on a pre-column which was then switched in-line to the analytical column for separation. Peptides were eluted with a gradient of acetonitrile in water/0.1% (v/v) formic acid (main step from 11-30.5% at a rate of 0.19% min^{-1}). The column was connected to a 10 µm SilicaTip™ nanospray emitter (New Objective, Woburn, MA, USA) for infusion into the mass spectrometer. Data analysis was performed using a CID/HCD fragmentation method with the following parameters: positive ion mode, orbitrap MS resolution = 60k, mass range (quadrupole) = 300-1800 m/z, MS2 in ion trap, threshold $2x10^4$, isolation window 1.6 Da, charge states 2-5, MS2 top20, AGC target $1.9x10^4$, max inject time 35 ms, dynamic exclusion 1 count, 15 s exclusion, exclusion mass window ±5 ppm. MS scans were saved in profile mode while MS2 scans were saved in centroid mode.

Raw files were processed with MaxQuant (version 1.6.1.0) (http://maxquant.org).[7] The peak lists were used to search against a custom database containing the proteins of interest in a background of 250 unrelated proteins using an in-house Mascot Server (2.4.1, Matrixscience, London, UK) with trypsin or AspN with 2 missed

cleavages and glutathionylation (Cys), oxidation (Met), acetylation (protein N-terminus), and deamidation (Asn,Gln) as variable modifications. Mass tolerances were 6 ppm for precursor ions and 0.6 Da for fragment ions.

In the second approach, an aliquot (250 μl) of the protein was treated with 20 μl of 0.54 M iodoacetamide, chloroform-methanol precipitated, and centrifuged at 17,000 × g for 5 min at an ambient temperature.[8] The upper aqueous methanol layer was removed, 500 μl of methanol added, gently mixed by inversion, and centrifuged as above. The supernatant was removed, and the pellet retained. Outside of the glove box, and prior to digestion, the pellet was dried at 37 °C for ~30 min. Digestion was carried out using the Proteoextract All-in-one Trypsin digestion kit (Calbiochem), following the manufacturer's *in-solution* (50 μl) digestion protocol, but omitting the reducing agent to preserve disulfide bonds. For double digests, the sample was first digested with trypsin (as described above), heat inactivated at 95 °C for ~10 min, then treated with AspN protease (New England Biolabs). All digestions were incubated over night at 37 °C. The resulting samples were diluted to 500 μl with 2% (v/v) acetonitrile 0.1% (v/v) formic acid and an aliquot (10 μl) of the sample was injected in to a Kinetic Evo C18 column (100 Å, 2.6 mm, 150 x 2.1 mm, Phenomenex) via an Ultimate 3000 uHPLC system (Dionex, Leeds, UK). Peptides were eluted (0.2ml/min) as follows: immediately after injection the organic phase was stepped from 2% to 10% (v/v) acetonitrile and held for 5 min, followed by a linear gradient between 10 and 100% acetonitrile, 0.1% (v/v) formic acid over 75 min. The eluent was continuously infused into a Bruker microQTOF-QIII mass spectrometer using positive mode electrospray ionisation (ESI); acquisition was controlled by Bruker oTOF Control software with the following parameters: dry gas flow of 8 L/min, nebulizer gas pressure of 0.8 Bar, dry gas at 200 °C, capillary voltage of 4,500 V, offset of 500 V, ion energy of 3 eV, and collision radio frequency of stepped between 150 and 600 Vpp. Nitrogen served as the carrier and collision gas. Ions ≥ 307 m/z and ≥1000 counts in intensity were selected for auto CID fragmentation; during this process the collision energy was swept between 70 - 150% (~25 to 70 eV) of default values. Bruker Smart exclusion was employed to reduce the selection background ions. HPLC and MS functions were integrated by Hystar (Bruker Daltonics, Coventry, UK)). Compass Data Analysis 4.1, Biotools and Sequence editor v3.2 (Bruker Daltonics, Coventry, UK)) were used for data processing.

Supplementary Tables

Table S1. Predicted and observed masses for different forms of NsrR.

**NsrR Species**	**Predicted mass (Da)**	**Average observed mass (Da)[a]**	**ΔMass (Da)[b]**
Apo-NsrR	15,954	15,953	-1
S^0	15,986	15,984	-2
Fe^{3+}	16,007	16,007	0
Iron Nitrosyls[c]			
$[Fe_2(NO)_4]^{2+}$	16,184	16,184	0
$[Fe_2(NO)_4(S^0)]^{2+}$	16,216	16,216	0
Thiol adducts[d]			
GS	16,259	16,259	0
$GS(S^0)$	16,291	16,291	0
$GS(S^0)$	16,323	16322	0
MS	16,438	16,438	0
$MS(S^0)$	16,470	16,470	0
$MS(S^0)_2$	16,502	16,502	0

[a]The average observed mass was derived from at least four independent experiments, with SD of ±1 Da.
[b]The difference between the average observed and predicted mass.
[c]Iron nitrosyl species formulae refer only to the non-protein components. Here, the predicted mass depends on the charge contributed by the iron-nitrosyl and the resulting charge compensation of iron-nitrosyl binding. The overall +2 charge is compensated for by coordination to deprotonated bridging thiolates (total =-2), giving an overall charge of zero for the protein-bound species. Because the thiolates are part of the protein, they are not shown as part of the adduct formulae here.
[d]Thiol adducts are covalent species connected by a disulfide bond. Hence the predicted mass equates to the neutral protein plus the neutral thiol minus two mass units due to loss of two protons.

Supplementary Figures

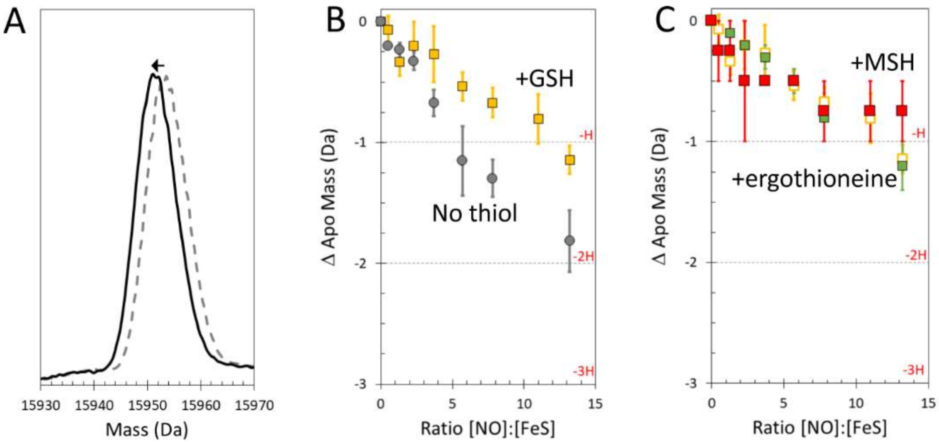

***Figure S1*. NO dependent disulfide bond formation in apo-NsrR. A)** Shift in the mass of the apo-NsrR monomer following reaction of [4Fe-4S] NsrR with nitric oxide. **B)** Plot of change of mass of apo-NsrR as a function of the NO to iron-sulfur cluster ratio in the presence (yellow squares) and absence (grey circles) of glutathione (GSH). During the course of the titration up two mass units are lost in an NO dependent manner, indicative of disulfide bond formation. The presence of GSH inhibits this process but does not abolish it. **C)** As in B) but with ergothioneine (green squares) or mycothiol (red squares) as the low molecular weight thiol. Data with GSH from B) are plotted (open yellow squares) for easy comparison. Samples were maintained under anaerobic conditions prior to LC-MS analysis. The data correspond to multiple repeats (n ≥2); error bars represent SEM.

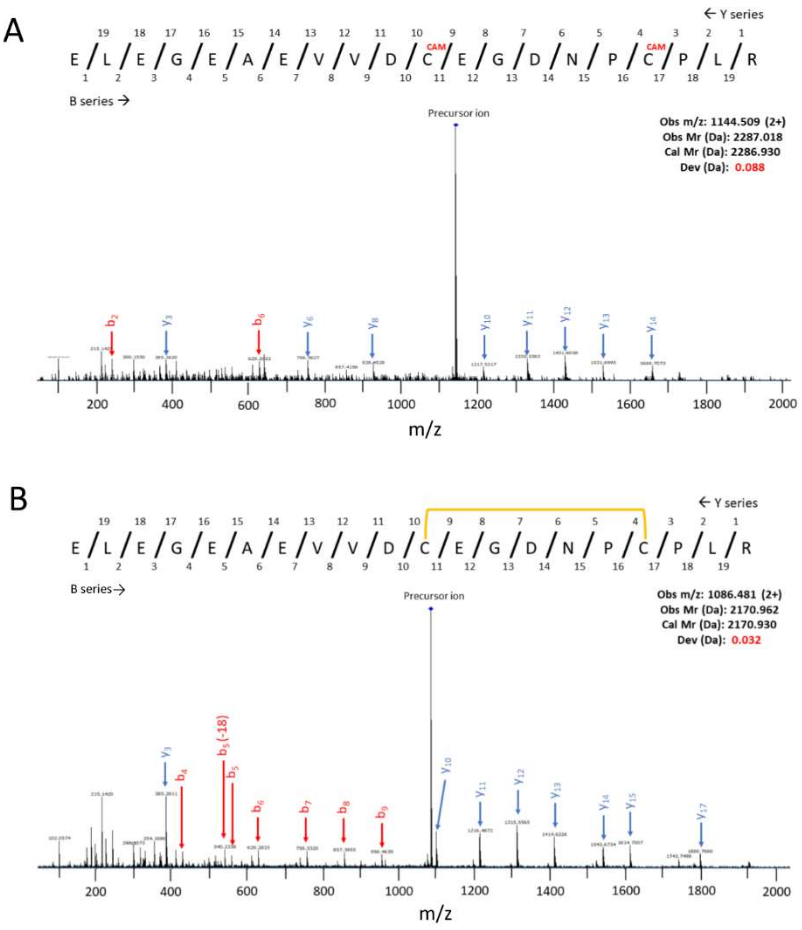

***Figure S2*. MS/MS analysis reveals the location of the disulfide bond of oxidised apo-NsrR. A)** MS/MS spectrum of a peptide fragment containing Cys93 and Cys99 residues, which are both carbomidomethylated (CAM) through reaction of the reduced thiolate forms with iodoacetamide. **B)** MS/MS of the same peptide fragment, but with the side chains of the two Cys residues in a disulfide bonded form (indicated by the yellow bridge line). B ions are in red, y ions are in blue. b_i (or y_i) indicates a singly charged ion corresponding to a fragment containing i residues. b_i (or y_i) (-18) indicates loss of water. Data acquired via a microQTOF-QIII (see Experimental procedures).

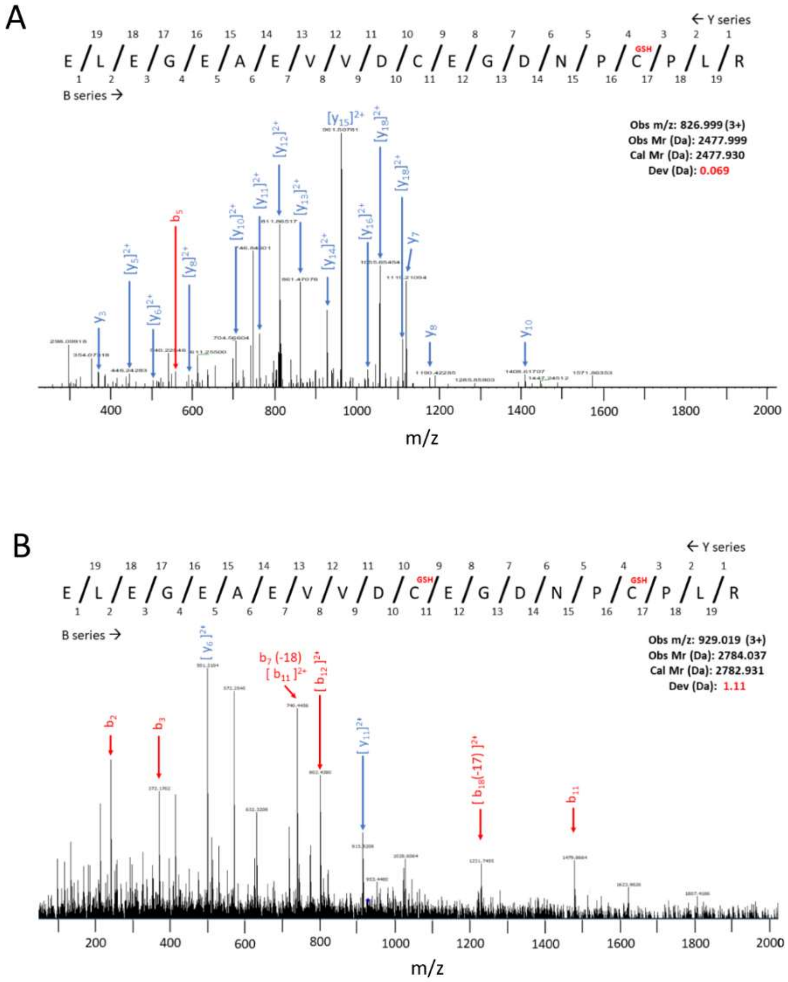

***Figure S3*. MS/MS analysis of GSH adducts of apo-NsrR. A)** MS/MS spectrum of a peptide fragment containing Cys93 and Cys99 residues, in which Cys99 is glutathionylated. **B)** MS/MS of the same peptide fragment, but with the side chains of both Cys93 and Cys99 glutathionylated. B ions are in red, y ions are in blue. b_i (or y_i) indicates a singly charged ion corresponding to a fragment containing i residues. b_i (or y_i) (-/+18) indicates loss or gain of water. b_i (or y_i) (-17) indicates loss of OH [b_i]$^{2+}$/ [y_i]$^{2+}$ indicates doubly charged ions. We note that the doubly glutathionylated species was not detected in standard LC-MS experiments, and we conclude that the additional sensitivity of the MS/MS experiment enabled the identification of the double GSH adduct peptide fragment. The data in A and B were acquired via an Orbitrap-Fusion™ or microQTOF-QIII, respectively (see Experimental procedures).

.

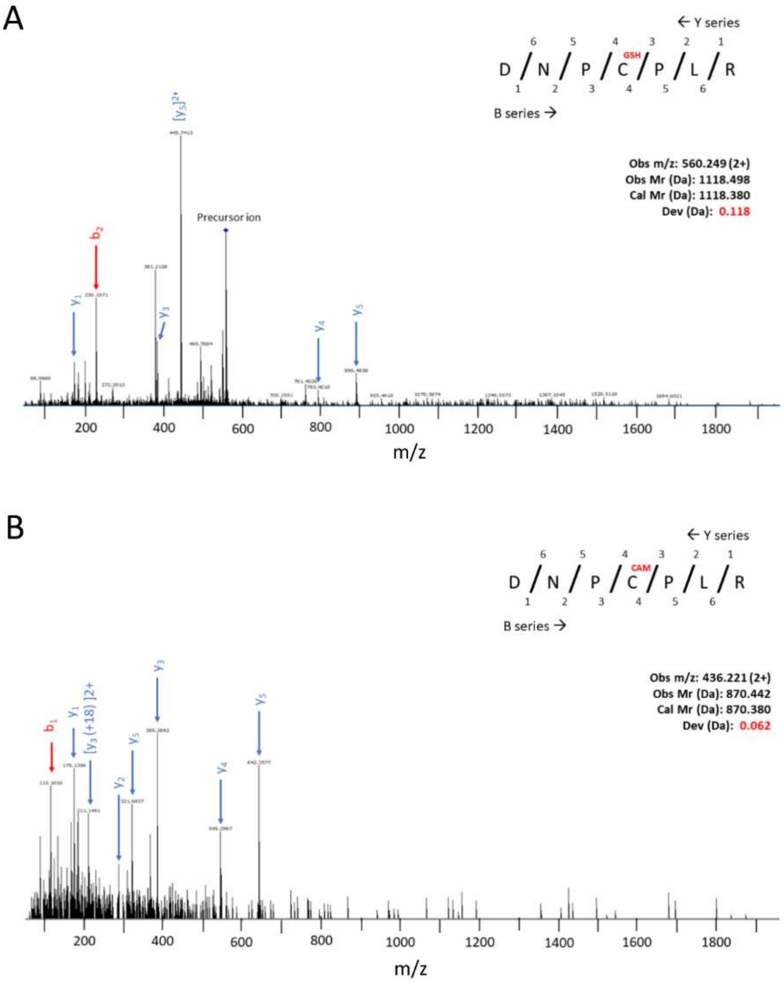

***Figure S4*. MS/MS analysis of a Cys99 GSH adduct of apo-NsrR. A)** MS/MS spectrum of a short peptide fragment, obtained by double digestion with trypsin and AspN, containing only one Cys residue (Cys99), which is glutathionylated. **B)** MS/MS of the same peptide fragment, but with the side chain of Cys99 in the carbomidomethylated (CAM) form following reaction of the reduced thiolate form with idoacetamide. B ions are in red, y ions are in blue. b_i (or y_i) indicates a singly charged ion corresponding to a fragment containing i residues. b_i (or y_i) (-/+18) indicates loss or gain of water. $[\ y_i\ ]^{2+}$ indicates doubly charged ions. Data acquired via microQTOF-QIII (see Experimental procedures).

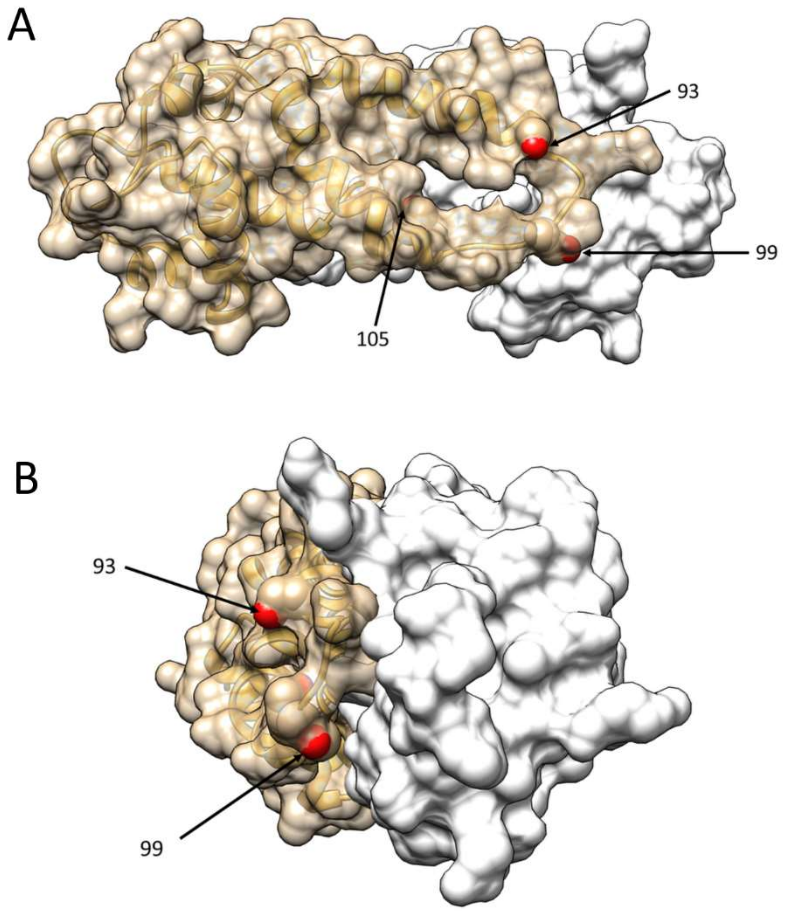

***Figure S5*. Structure of apo-NsrR showing the positions of residues 93, 99 and 105. A)** Front and **B)** side view of the cluster-free NsrR dimer, with one protomer in beige and the other in grey/white. The structure is shown in a surface representation with partial transparency of the beige protomer to enable visualisation of the protein backbone. The structure was generated from a triple variant of NsrR in which the three conserved Cys residues, Cys93, 99 and 105, were replaced by serines.[9] These residues are shown in space space filled mode and labelled according to residue number. The figures were generated using PDB ID: 5N08 [9] and USCF Chimeria.[10]

References

1. J. C. Crack, J. Munnoch, E. L. Dodd, F. Knowles, M. M. Al Bassam, S. Kamali, A. A. Holland, S. P. Cramer, C. J. Hamilton, M. K. Johnson, A. J. Thomson, M. I. Hutchings and N. E. Le Brun, *J. Biol. Chem.*, 2015, **290**, 12689-12704.
2. P. K. Smith, R. I. Krohn, G. T. Hermanson, A. K. Mallia, F. H. Gartner, M. D. Provenzano, E. K. Fujimoto, N. M. Goeke, B. J. Olson and D. C. Klenk, *Anal. Biochem.*, 1985, **150**, 76-85.
3. J. C. Crack, J. Green, A. J. Thomson and N. E. Le Brun, *Meth.Mol. Biol.*, 2014, **1122**, 33-48.
4. P. N. Serrano, H. Wang, J. C. Crack, C. Prior, M. I. Hutchings, A. J. Thomson, S. Kamali, Y. Yoda, J. Zhao, M. Y. Hu, E. E. Alp, V. S. Oganesyan, N. E. Le Brun and S. P. Cramer, *Angew. Chemie*, 2016, **55**, 14575-14579.
5. S. V. Sharma, K. Van Laer, J. Messens and C. J. Hamilton, *Chembiochem*, 2016, **17**, 1689-1692.
6. P. W. Riddles, R. L. Blakeley and B. Zerner, *Meth. Enzymol.*, 1983, **91**, 49-60.
7. S. Tyanova, T. Temu and J. Cox, *Nat. Protocols*, 2016, **11**, 2301-2319.
8. D. B. Friedman and K. S. Lilley, in Protein protocols (J. M. Walker, Ed), 3rd Edition, Humana Press, 2009, pp225-227.
9. A. Volbeda, E. L. Dodd, C. Darnault, J. C. Crack, O. Renoux, M. I. Hutchings, N. E. Le Brun and J. C. Fontecilla-Camps, *Nat. Commun.*, 2017, **8**, 15052.
10. E. F. Pettersen, T. D. Goddard, C. C. Huang, G. S. Couch, D. M. Greenblatt, E. C. Meng and T. E. Ferrin, *J. Comput. Chem.*, 2004, **25**, 1605-1612.
